# Polymorphisms of Homologous Recombination Genes and Clinical Outcomes of Non-Small Cell Lung Cancer Patients Treated with Definitive Radiotherapy

**DOI:** 10.1371/journal.pone.0020055

**Published:** 2011-05-25

**Authors:** Ming Yin, Zhongxing Liao, Yu-Jing Huang, Zhensheng Liu, Xianglin Yuan, Daniel Gomez, Li-E Wang, Qingyi Wei

**Affiliations:** 1 Department of Epidemiology, The University of Texas M. D. Anderson Cancer Center, Houston, Texas, United States of America; 2 Department of Radiation Oncology, The University of Texas M. D. Anderson Cancer Center, Houston, Texas, United States of America; 3 Department of Oncology, Tongji Hospital, Tongji Medical College, Huazhong University of Science and Technology, Wuhan, China; National Cancer Institute, United States of America

## Abstract

The repair of DNA double-strand breaks (DSBs) is the major mechanism to maintain genomic stability in response to irradiation. We hypothesized that genetic polymorphisms in DSB repair genes may affect clinical outcomes among non-small cell lung cancer (NSCLC) patients treated with definitive radio(chemo)therapy. We genotyped six potentially functional single nucleotide polymorphisms (SNPs) (i.e., *RAD51* −135G>C/rs1801320 and −172G>T/rs1801321, *XRCC2* 4234G>C/rs3218384 and R188H/rs3218536 G>A, *XRCC3* T241M/rs861539 and *NBN* E185Q/rs1805794) and estimated their associations with overall survival (OS) and radiation pneumonitis (RP) in 228 NSCLC patients. We found a predictive role of *RAD51* −135G>C SNP in RP development (adjusted hazard ratio [HR] = 0.52, 95% confidence interval [CI], 0.31–0.86, *P* = 0.010 for CG/CC vs. GG). We also found that R*AD51* −135G>C and *XRCC2* R188H SNPs were independent prognostic factors for overall survival (adjusted HR = 1.70, 95% CI, 1.14–2.62, *P* = 0.009 for CG/CC vs. GG; and adjusted HR = 1.70; 95% CI, 1.02–2.85, *P* = 0.043 for AG vs. GG, respectively) and that the SNP-survival association was most pronounced in the presence of RP. Our study suggests that HR genetic polymorphisms, particularly *RAD51* −135G>C, may influence overall survival and radiation pneumonitis in NSCLC patients treated with definitive radio(chemo)therapy. Large studies are needed to confirm our findings.

## Introduction

Lung cancer leads all other cancers in both incidence and mortality worldwide. Non-small cell lung cancer (NSCLC) accounts for 89% of all lung cancer, and most patients have advanced stages at diagnosis, requiring radiotherapy alone or in combination with chemotherapy to improve the local control and overall survival. However, despite aggressive treatment in these patients, the prognosis is still unsatisfactory, with a 5-year survival rate of about 10% [1] and a median survival time (MST) of 16–18 months [2]. Meanwhile, radiation treatment-related pulmonary toxicity, such as pneumonitis and pulmonary fibrosis, may influence the prognosis of NSCLC patients, because these complications restrict the dose of radiation used and compromise pulmonary functions. Therefore, there has been a persistent interest in search for readily accessible molecular markers that may help assess therapeutic benefits by predicting clinical outcomes of those NSCLC patients who are treated with definitive radio(chemo)therapy.

The DNA double-strand breaks (DSBs) are the principle genotoxic lesions of ionizing radiation, which pose major threats to genomic integrity. It is estimated that a dose of ∼1 Gy of X-rays produces about 50–100 double-strand breaks in the DNA of a typical mammalian cell, leading to 50% cell death [3], [4]. There are two main pathways for DNA DSB repair—homologous recombination (HR) and non-homologous end-joining (NHEJ). In HR, the initial step involves recognition and signaling of DSB by a protein complex of NBN, MRE11 and RAD50; then RAD51 protein is recruited to catalyze the strand exchange reaction and some RAD51-related proteins, such as RAD51 B-D, XRCC2 and XRCC3, participate in the assembly of the RAD51 nucleoprotein filament and the selection and interaction with the appropriate recombination substrates [5].

There is a genetic basis of cellular responses to ionizing radiation in cancer treatment, because patients receiving similar treatment could have different response to radiotherapy. Previous studies demonstrated that elevated expression of some DSB repair proteins, such as RAD51, NBN and XRCC3, confers radioresistance [6], [7], [8], whereas loss of XRCC2 results in a severe delay in the early response of DSB [9]. Patients of a rare congenital disorder, Nijmegen breakage syndrome, are extremely sensitive to radiation because of a compromised DSB repair capacity due to *NBN* mutations [10]. Hence, it is reasonable to speculate that the inter-individual variability in DSB repair capacity may modulate phenotype of radiosensitivity and clinical outcomes of radiotherapy.

Since single nucleotide polymorphisms (SNPs) may modify gene function or can be used as genetic markers to detect nearby disease-causing variants through association or linkage studies, we sought to evaluate the association of six potentially functional SNPs (i.e., *RAD51* −135G>C/rs1801320, −172G>T/rs1801321, *XRCC2* 4234G>C/rs3218384, R188H/rs3218536 G>A, *XRCC3* T241M/rs861539, and *NBN* E185Q/rs1805794) of genes involved in HR pathway with radiation pneumonitis (RP) and overall survival (OS) of NSCLC patients treated with definitive radio(chemo)therapy in the present study.

## Materials and Methods

### Ethics statement

This study was approved by The University of Texas M. D. Anderson Cancer Center Institutional Review Board and informed consents were waived. We complied with HIPAA regulations.

### Study populations

In the study, clinical data were derived from a dataset of 261 patients with histopathologically confirmed NSCLC, who were treated with definitive radiation at our institution between 1999 and 2005. Among these 261 patients, 231 patients had documented survival information. After we excluded those patients who had surgical resection or recurrence before radiotherapy, the final data pool consisted of 228 patients with stage IA to IV NSCLC and survival information and 196 patients with pneumonitis information and radiation dosimetric data. The details of the radiation treatment planning, follow-up schedule and tests, and dosimetric data analysis were described in our previous publication [11].

### Selection of SNPs and genotyping

We selected six common (minor allele frequency >0.05 in Caucasians), well-studied functional variants of *RAD51*, *XRCC2*, *XRCC3* and *NBN* genes involved in HR pathway: *RAD51* −135G>C/rs1801320, −172G>T/rs1801321, *XRCC2* 4234G>C/rs3218384, R188H/rs3218536 G>A, *XRCC3* T241M/rs861539 and *NBN* E185Q/rs1805794. This is because they are located in promoter region or cause nonsynonymous amino acid changes that have been reportedly associated with cancer risk or survival. Although *MRE11* and *RAD50* genes are also important in the initial stage of response to DSB, no functional SNPs have been reported so far and therefore, these two genes were not included in our study. Genomic DNA was extracted from the buffy coat fraction of each blood sample by using a Blood Mini Kit (Qiagen, Valencia, CA) according to the manufacturer's instructions. DNA purity and concentrations were determined by spectrophotometric measurement of absorbance at 260 and 280 nm by a UV spectrophotometer (Nano Drop Technologies, Inc., Wilmington, DE). Genotypes were generated by the polymerase chain reaction (PCR)-restriction fragment length polymorphism (RFLP) method (Figure S1). The primer sequences, restriction enzymes and PCR conditions used for the experiments were available upon request.

### Selection of end points

We selected overall survival and occurrence of radiation pneumonitis as our study end points. There are five grades of radiation-treatment related pneumonitis according to the Common Terminology Criteria for Adverse Events version 3.0 [12]. Previous studies have used two different types of criterion in RP investigation: RP of any grade (grade≥1) [13], [14], [15] and RP requiring clinical intervention (grade≥2 or 3) [16], [17]. In this study, we used RP of any grade (grade≥1) to include more RP events. All times to the endpoints were calculated from the first day of radiation treatment until the date of event or the last known follow-up.

### Statistical methods

The hazard ratios (HRs) with their corresponding 95% confidence intervals (CIs) were calculated by Cox proportional hazard analyses to evaluate the influence of various genotypes on overall survival and radiation pneumonitis. The HRs for OS were adjusted for age, sex, race, Karnofsky performance scores (KPS), smoking status, tumor histology, disease stage, application of chemotherapy and radiation dose; whereas the HRs for RP were adjusted for age, sex, race, KPS, smoking status, tumor histology, disease stage, application of chemotherapy and mean lung dose because mean lung dose is the traditional dosimetric factor to be associated with RP. Kaplan-Meier analysis was used to evaluate the effect of different genotypes on the cumulative probability of overall survival and radiation pneumonitis. All reported *P* values were two-sided, and *P*<0.05 indicates statistical significance. All analyses were performed using SAS software (version 9.1; SAS Institute, Cary, NC). The Bonferroni method was used to adjust for multiple comparisons.

## Results

### Population characteristics

The 228 patients used for the final analysis included 125 men and 103 women, with a median age of 63 years (range, 35 to 88 years). Among them, 74.6% were white, 82.5% had stage III/IV disease, 88.2% were treated with a combination of chemotherapy and radiotherapy, and 96.8% received radiation doses between 60 and 70 Gy. The median overall survival time was 20 months and the median time for RP development was 4.3 months. Patient-, disease-, and treatment-related characteristics and their influences on OS and RP (grade≥1) are shown in Table 1. In the univariate analysis, we found that male, tumor stage of III/IV, and histology of “not otherwise specified” were significantly associated with reduced OS, whereas tumor stage of I/II seemed to be associated with reduced RP. The most commonly used dosimetric factor of mean lung dose was only available for the 196 patients who were treated with 3-dimensional conformal radiation and who had pneumonitis information and was marginally associated with RP development in our analysis (Table 1).

**Table 1:** Patient demographics and association with clinical outcomes of overall survival (OS) and radiation pneumonitis (RP).

	OS (N = 228)				RP (grade≥1, N = 196)			
Parameter	No. (%)	HR	95% CI	*P* †	No. (%)	HR	95% CI	*P* †
Sex								
Female	103 (45.2)	1.00			83 (42.4)	1.00		
Male	125 (54.8)	1.51	1.08–2.11	**0.017**	113 (57.6)	1.09	0.78–1.54	0.608
Age (years)								
<63	113 (49.6)	1.00			97 (49.5)	1.00		
≥63	115 (50.4)	1.37	0.96–1.94	0.080	99 (50.5)	1.02	0.73–1. 43	0.900
Race								
White	170 (74.6)	1.00			141 (71.9)	1.00		
Black	45 (19.7)	0.96	0.60–1.54	0.875	42 (21.4)	1.21	0.81–1.81	0.360
Other	13 (5.7)	1.51	0.73–3.12	0.269	13 (6.7)	1.11	0.54–2.28	0.776
KPS								
<80	59 (25.9)	1.00			40 (20.4)	1.00		
≥80	169 (74.1)	0.71	0.46–1.11	0.133	156 (79.6)	1.02	0.61–1.71	0.934
TNM stage§								
III, IV	188 (82.5)	1.00			167 (85.2)	1.00		
I, II	34 (14.9)	0.55	0.32–0.94	**0.029**	29 (14.8)	0.55	0.33–0.93	**0.026**
Histology§								
Adenocar	77 (33.8)	1.00			66 (33.7)	1.00		
NSCLC, NOS	74 (32.5)	1.56	1.04–2.33	**0.032**	67 (34.2)	0.99	0.66–1.47	0.949
Squamous cell	70 (30.7)	1.41	0.92–2.14	0.138	63 (32.1)	0.73	0.48–1.12	0. 471
Smoking status								
Ever	200 (87.7)	1.00			177 (90.8)	1.00		
Never	28 (12.3)	1.07	0.61–1.89	0.798	18 (9.2)	1.36	0.77–2.40	0.292
Chemotherapy§								
No	22 (9.6)	1.00			20 (10.3)	1.00		
Yes	201 (88.2)	1.02	0.54–1.91	0.957	175 (89.7)	1.61	0.84–3.07	0.151
Mean lung dose (Gy)								
<17.67					57 (29.1)	1.00		
≥17.67					139 (70.9)	1.40	0.95–2.04	0.087
Radiation dose (Gy)								
<63	46 (20.2)	1.00			36 (18.4)	1.00		
≥63	182 (79.8)	0.79	0.50–1.21	0.272	160 (81.6)	0.73	0.47–1.13	0.154

† *P* values were calculated by univariate Cox proportional hazards model.

§ The numbers for certain variables may not add up to the total number because of the missing information.

NOS: Not otherwise specified; KPS: Karnofsky Performance Score.

### Association between overall survival and polymorphisms

**Figure 1:**
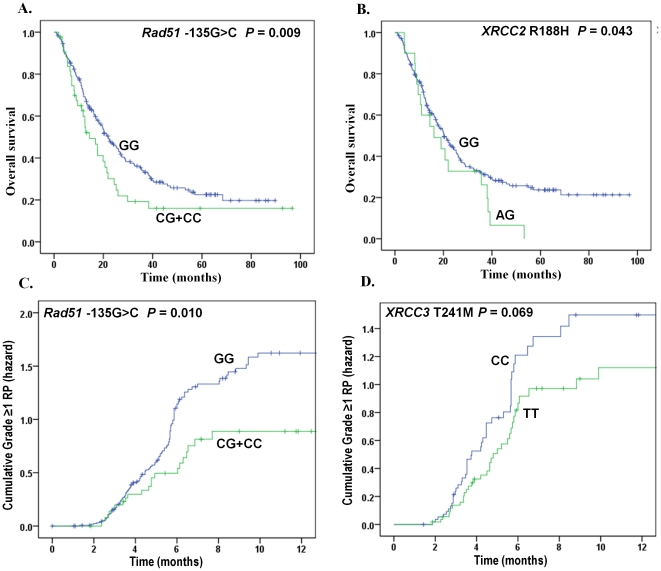
Overall survival and risk of radiation pneumonitis by selected polymorphisms of DNA double-strand break genes. (A) and (B), overall survival by *RAD*51 −135G>C and *XRCC2* R188H SNPs; (C) and (D), radiation pneumonitis by *RAD*51 −135G>C and *XRCC3* T241M SNPs. The *P* values were obtained from the Cox hazards model with adjustment.

The genotype distributions of SNPs in genes involved in the HR pathway and the association with OS are summarized in Table 2. Compared with the homozygote *RAD51* −135GG genotype, the CG/CC genotypes were marginally associated with a hazard of early death in the univariate analysis (HR = 1.46, 95% CI, 0.99–2.14, *P* = 0.056). After a multivariate adjustment for age, sex, smoking status, tumor histology, KPS, tumor stage, application of chemotherapy, and radiation dose, the HRs were statistically significant in both *RAD51* −135G>C and *XRCC2* R188H SNPs (CG/CC vs. GG: adjusted HR = 1.70; 95% CI, 1.14–2.62, *P* = 0.009; and AG vs. GG: adjusted HR = 1.70; 95% CI, 1.02–2.85, *P* = 0.043, respectively) (Table 2 and Fig. 1A, 1B).

**Table 2:** Univariate and multivariate analyses of different genotypes and OS for NSCLC (N = 228).

Genotypes	Patient No.	Event	Crude		*P* *	Adjusted		*P* †
			HR	95% CI		HR	95% CI	
*RAD51*								
(rs1801320 −135G>C)								
GG	183	124	1.00			1.00		
CG	39	29	1.53	1.02–2.30	**0.040**	1.71	1.11–2.64	**0.014**
CC	5	4	1.07	0.39–2.88	0.900	1.52	0.54–4.24	0.429
CG+CC	44	33	1.46	0.99–2.14	0.056	1.70	1.14–2.62	**0.009**
*RAD51*								
(rs1801321 −172G>T)								
TT	144	100	1.00			1.00		
TG	84	58	1.14	0.82–1.58	0.429	1.03	0.73–1.45	0.879
GG	0	0	N/A			N/A		
TG+GG	84	58	1.14	0.82–1.58	0.429	1.03	0.73–1.45	0.879
*XRCC2*								
(rs3218384 4234G>C)								
GG	137	97	1.00			1.00		
GC	78	53	0.77	0.55–1.08	0.125	0.81	0.56–1.17	0.260
CC	13	8	0.63	0.30–1.29	0.202	0.61	0.28–1.35	0.221
GC+CC	91	61	0.75	0.54–1.03	0.075	0.76	0.54–1.09	0.135
*XRCC2*								
(rs3218536 G>A R188H)								
GG	205	138	1.00			1.00		
AG	20	18	1.44	0.88–2.36	0.144	1.70	1.02–2.85	**0.043**
AA	0	0	N/A			N/A		
AG+AA	20	18	1.44	0.88–2.36	0.144	1.70	1.02–2.85	**0.043**
*XRCC3*								
(rs861539 C>T T241M)								
CC	71	46	1.00			1.00		
CT	98	73	1.22	0.84–1.77	0.297	1.17	0.79–1.72	0.428
TT	59	39	0.91	0.60–1.40	0.681	0.89	0.57–1.38	0.593
CT+TT	157	112	1.09	0.78–1.54	0.613	1.06	0.74–1.51	0.766
*NBN*								
(rs1805794 E185Q)								
CC	109	71	1.00			1.00		
CG	98	72	1.04	0.75–1.44	0.832	1.06	0.75–1.51	0.735
GG	20	14	1.06	0.60–1.87	0.850	0.89	0.48–1.65	0.707
CG+GG	118	86	1.08	0.79–1.47	0.653	1.06	0.76–1.49	0.720

*P values were calculated by Cox proportional model using univariate analysis.

† *P* values were calculated with adjustment for age, sex, smoking status, tumor histology, KPS score, tumor stage, application of chemotherapy and radiation dose.

### Association between radiation pneumonitis and polymorphisms

The distributions of genotypes and their association with RP (grade≥1) are summarized in Table 3. Similar to the data of OS, only *RAD51* −135G>C SNP showed a significant association with RP in the univariate analysis (CG/CC vs. GG: HR = 0.56, 95% CI, 0.35–0.90, *P* = 0.017). After a multivariate adjustment, the significance still remained for *RAD51* −135G>C SNP in the dominant model (CG/CC vs. GG: HR = 0.52, 95% CI, 0.31–0.86, *P* = 0.010). The TT genotype of *XRCC3* T241M SNP showed a marginally protective effect against RP in the additive model (TT vs. CC: HR = 0.63, 95% CI, 0.38–1.04, *P* = 0.069) (Table 3 and Fig. 1C, 1D).

**Table 3:** Univariate and multivariate analyses of different genotypes and RP (grade≥1) for NSCLC (N = 196).

Genotypes	Patient No.	Event	Crude		*P* *	Adjusted		*P* †
			HR	95% CI		HR	95% CI	
*RAD51*								
(rs1801320 −135G>C)								
GG	156	115	1.00			1.00		
CG	34	18	0.55	0.34–0.91	**0.020**	0.50	0.29–0.84	**0.009**
CC	5	3	0.60	0.15–2.44	0.477	0.80	0.19–3.37	0.764
CG+CC	39	21	0.56	0.35–0.90	**0.017**	0.52	0.31–0.86	**0.010**
*RAD51*								
(rs1801321 −172G>T)								
TT	125	89	1.00			1.00		
TG	71	48	0.82	0.58–1.17	0.279	0. 730	0.50–1.08	0.117
GG	0	0	N/A			N/A		
TG+GG	71	48	0.82	0.58–1.17	0.279	0. 730	0.50–1.08	0.117
*XRCC2*								
(rs3218384 4234G>C)								
GG	120	85	1.00			1.00		
GC	66	47	1.03	0.72–1.47	0.870	1.21	0.81–1.80	0.361
CC	10	5	0.86	0.35–2.13	0.748	0.90	0.35–2.32	0. 830
GC+CC	76	52	1.01	0.72–1.43	0.949	1.17	0.79–1.73	0.437
*XRCC2*								
(rs3218536 G>A R188H)								
GG	173	126	1.00			1.00		
AG	16	8	0.56	0.27–1.14	0.107	0.55	0.25–1.19	0.129
AA	0	0	N/A			N/A		
AG+AA	16	8	0.56	0.27–1.14	0.107	0.55	0.25–1.19	0.129
*XRCC3*								
(rs861539 C>T T241M)								
CC	58	43	1.00			1.00		
CT	81	59	0.90	0.61–1.33	0.591	0.92	0.60–1.40	0.690
TT	57	35	0.70	0.45–1.09	0.119	0.63	0.38–1.04	0.069
CT+TT	138	94	0.81	0.57–1.17	0.263	0.80	0.54–1.19	0.271
*NBN*								
(rs1805794 E185Q)								
CC	93	68	1.00			1.00		
CG	85	57	0.98	0.69–1.39	0.909	1.09	0.75–1.60	0.651
GG	16	11	1.12	0.59–2.13	0.722	1.09	0.54–2.19	0.805
CG+GG	101	68	1.00	0.71–1.40	1.000	1.10	0.76–1.58	0.629

*P values were calculated by Cox proportional model using univariate analysis.

† *P* values were calculated with adjustment for age, sex, smoking status, tumor histology, KPS score, tumor stage, application of chemotherapy and mean lung dose.

### Modification effects of RP by SNPs of *RAD51* −135G>C and *XRCC2* R188H on overall survival

**Figure 2:**
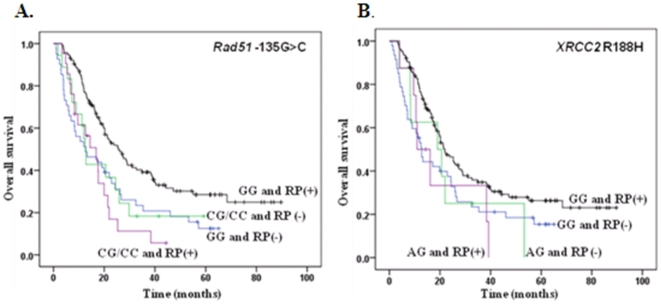
Overall survival by selected polymorphisms of DNA double-strand break genes according to the presence or absence of RP.

Since *RAD51* −135G>C and *XRCC2* R188H SNPs were also independent prognostic factors of overall survival, we then investigated the modification effect of RP by SNPs on OS. We found that both *RAD51* −135G>C and *XRCC2* R188H SNPs showed remarkable influence on OS only in the presence of RP (adjusted HR = 3.03. 95% CI, 1.69–5.45, *P*<0.001; and adjusted HR = 2.67. 95% CI, 1.13–6.29, *P* = 0.025, respectively) (Table 4 and Fig. 2). There was no difference in the death hazard between genotypes if RP was absent.

**Table 4:** *RAD51* −135G>C and *XRCC2* R188H genotypes and OS by RP (N = 196).

Genotypes	Patient No.	Event	Crude		*P* *	Adjusted		*P* †
			HR	95% CI		HR	95% CI	
*RAD51*								
(rs1801320 −135G>C)								
Absence of RP								
GG	41	35	1.00			1.00		
CG+CC	18	14	0.93	0.50–1.73	0.817	0.81	0.39–1.68	0.575
Presence of RP								
GG	115	73	1.00			1.00		
CG+CC	21	18	2.45	1.45–4.14	0.001	3.03	1.69–5.45	**<0.001**
*XRCC2*								
(rs3218536 G>A R188H)								
Absence of RP								
GG	47	38	1.00			1.00		
AG	8	7	1.02	0.45–2.29	0.889	1.22	0.51–2.92	0.653
Presence of RP								
GG	126	83	1.00			1.00		
AG	8	7	1.98	0.92–4.30	0.080	2.67	1.13–6.29	**0.025**

*P values were calculated by Cox proportional model using the univariate analysis.

† *P* values were calculated with adjustment for age, sex, smoking status, tumor histology, KPS score, tumor stage, application of chemotherapy and radiation dose.

### Bonferroni corrections

Because multiple testing may have been contributed to the findings in the evaluation of the statistical significance of our primary results, we also considered adjustment for the associations with the outcomes. After Bonferroni corrections, only *RAD51* −135G>C SNP remained significant for its association with both OS and RP.

## Discussion

To our knowledge, this is the first study to investigate the association between clinical outcomes and potentially functional polymorphisms of genes in the HR pathway in NSCLC patients treated with radiotherapy, with a reasonable sample size (a total of 228). We provided a strong evidence of the predictive value of *RAD51* −135G>C SNP for RP development. R*AD51* −135G>C and *XRCC2* R188H SNPs were also independent prognostic factors of overall survival, and the SNP-survival association was most pronounced in the presence of RP.

Lung cancer is a disease resulting from interactions between genetic and environmental factors, and its progression and prognosis are also heavily influenced by patient host or treatment-related factors. Some well-known prognostic factors in inoperable NSCLC patients, such as being male, old age and advanced tumor stage [18], were present in the current study. Although the mean lung dose was marginally associated with RP development by the criterion of grade≥1, it was significantly associated with grade≥3 RP (data not shown), which was consistent with our previous report [19]. The most important finding from the current study, however, is the modification effect of RP by HR genetic polymorphisms on OS in NSCLC patients receiving radio(chemo)therapy.

Current models of HR-mediated DSB repair suggest that DSBs are first recognized and bound by the MRE11-RAD50-NBN (MRN) complex. MRE11 has both endonuclease and exonuclease activities that are important for DNA end processing, and RAD50 can bind to DNA that may be involved in tethering sister chromatids, whereas NBN forms the flexible adapter domain of MRN and provides the MRN complex with its signaling role through interactions and activation of its downstream proteins that ultimately activate the RAD51-dependent HR pathway [20].

The *RAD51* gene family consists of several proteins that show DNA-stimulated ATPase activity and plays a central role in the HR activation. It interacts directly with several repair proteins, including XRCC2, XRCC3, BRCA1/2, to form a complex essential for repair of DNA cross-links (especially XRCC2 and XRCC3), which maintains genomic stability by using the sister chromatid as a template for precise repair. Previous studies found some potentially functional SNPs of HR genetic polymorphisms (e.g., *RAD51*-135 G>C, −172G>T, *XRCC2* 4234G>C, R188H, *XRCC3* T241M and *NBN* E185Q) to be associated with various types of cancer risks, including cancers of the lung, breast, ovary, leukemia and head and neck [21]. Some SNPs also showed a significant effect on survival outcomes, such as *RAD51*-135 G>C in breast cancer [22], *XRCC2* R188H in pancreatic cancer [23], and SNPs of *RAD51*-135 G>C and *XRCC3* T241M in acute myeloid leukemia [24]. However, most studies performed in prostate or breast cancer failed to find any significant association with risk of radiation-induced complications [25], [26]. In the current study, there appeared a significant SNP-survival association (*XRCC2* R188H) and a marginally significant SNP-RP association (*XRCC3* T241M) in radiation-treated NSCLC patients. Most importantly, the SNP of the central HR gene, *RAD51* −135G>C SNP, was robustly associated with both OS and RP after Bonferroni corrections. This result was confirmed by receiver operating characteristic (ROC) curves analyses, which showed that *RAD51* −135G>C in particular was highly valuable to improve the predictive power of assessing clinical outcomes (e.g., OS and RP) of NSCLC patients treated with radiotherapy (data not shown). Taken together, our results demonstrated the importance of the HR pathway in response to thoracic radiation and possible tumor-specific influence from HR genetic polymorphisms.

To date, the functional changes of *RAD51*, *XRCC2*, *XRCC3* and *NBN* caused by the SNPs included in the current study have not been well studied. Some studies found that the threonine-to-methionine substitution at codon 241 of the *XRCC3* gene (T241M) was associated with radiosensitivity in non-cancer subjects but did not cause significant difference in DNA repair capacity between the variant and wild-type genotypes [27], [28], while the glutamine-to-glutamic acid substitution at codon 185 of *NBN* gene (E185Q) may influence the interaction between NBN and BRCA1 proteins responsible for recognition and repair of aberrant DNA [29], [30]. However, the role of arginine-to-histidine substitution at codon 188 of the *XRCC2* gene (R188H) is largely unknown. The −135 G>C SNP, located in the promoter region of the *RAD51* gene, results in the up-regulated gene expression through an increased promoter activity by substituting G for C allele [31]. RAD51 is essential for optimal repair of DSBs, and its expression level was a single important factor in modifying DSB repair capacity as reported in previous studies [32], [33]. Based on these findings, the association of the *RAD51* −135C allele with a decreased hazard for RP and a reduced OS can be reasonably explained by an increased radioresistance due to the anticipated up-regulated RAD51 expression. Although the functional relevance of *XRCC2* R188H with clinical outcomes of NSCLC patients has not been clarified, it may either result from its effect on the gene functions or its linkage with other functional SNPs, which need to be unraveled by additional mechanistic studies.

The relationship among RP, OS and SNPs of NSCLC patients was complex and could only be explored preliminarily in the current investigation. Inoue *et al.* reported severe RP (grade 3–4) to be an adverse prognostic factor [34], whereas our study showed *RAD51* G>C to be associated with a reduced RP incidence but a decreased OS. This is not contradictory, because our RP group included a large proportion of patients with mild/moderate RP (grade 1 and 2), which was not likely to have an impact on patients' survival negatively. Furthermore, some other studies also did not find any association between RP grade and prognosis [35].

In this study, we were much interested in the finding that the influences of *RAD51* −135G>C and *XRCC2* R188H on overall survival were only evident in patients with RP. Several possibilities may explain these findings. First, irradiation of lung tissues induces immediate damage through intracellular protein denaturation, membrane disruption, and alterations of DNA. RP is largely a consequence of cellular DNA injury that appears in the second generation of cells [36]. The absence of RP may imply a low amount of DNA injury, which could result from an efficient DNA repair, whereas the presence of RP may indicate a high amount of DNA injury due to a suboptimal DNA repair. Therefore, it can be speculated that if the host cells have optimal DNA repair capacity, the activity of the entire HR machinery is less likely to be influenced by a single HR genetic polymorphism. On the contrary, if the DNA repair capacity is suboptimal, the subsequent changes in gene expression or activity resulting from functional SNPs may pose a substantial influence on the whole HR machinery. Second, RP *per se* is an inflammatory response to ionizing radiation, which triggers a large network of signaling events, including transient activation of pro-survival pathways such as the epidermal growth factor receptor (EGFR) pathway [37], and upregulation of a variety of cytokines, such as tumor necrosis factor alpha (TNF-α), interleukins, and transforming growth factor beta (TGF-β) [38], [39], [40]. Although many of these inflammatory responses are harmful to normal tissue, they confer a survival advantage of tumor cells and regulate cellular radiation response and DNA repair. Hence, the SNP-survival association could be more pronounced in the presence of RP. Additional mechanistic studies are needed to test these hypotheses.

Despite these positive findings, our study has some limitations. First, we were not able to explore the mechanism of how the HR genetic polymorphisms and RP influence survival outcomes of lung cancer patients. Secondly, we used a candidate polymorphism approach, which allowed us to focus on potentially functional SNPs reported in the literatures but did not comprehensively cover all SNPs in the entire gene. Some important SNPs may have been missed or the observed association may result from genetic linkages with other untyped SNPs. These warrant additional investigation of the tagging SNPs that may help find the underlying disease-causing variants in future studies. Thirdly, our sample size is still not large enough to perform stratified analyses for identifying the high-risk subgroups.

In summary, we found that HR genetic polymorphisms, particularly *RAD51* −135G>C, may influence the overall survival and risk of radiation pneumonitis in NSCLC patients treated with definitive radio(chemo)therapy. Future prospective studies with large sample sizes and better study designs are required to confirm our findings.

## Supporting Information

Figure S1 **PCR-based restriction analysis.** Genotypes of the *RAD51*, *XRCC2*, *XRCC3* and *NBN* SNPs were shown on agarose electrophoresis.
(TIF)
