# Supplementary figures and images for: Polymorphisms of Homologous Recombination Genes and Clinical Outcomes of Non-Small Cell Lung Cancer Patients Treated with Definitive Radiotherapy
Source: PLoS One. 2011 May 25;6(5):e20055. doi: 10.1371/journal.pone.0020055 (PMC3102071; doi:10.1371/journal.pone.0020055)

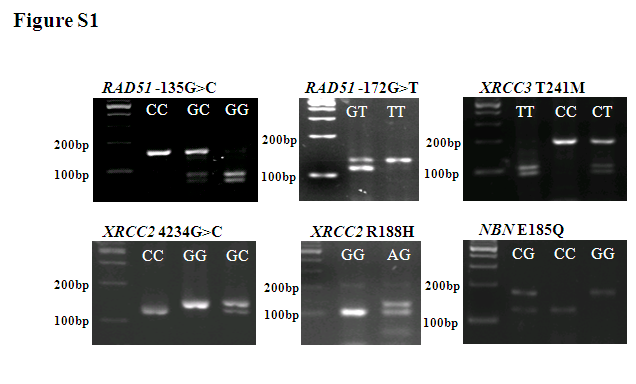

Supplement: Figure S1 — PCR-based restriction analysis. Genotypes of the RAD51, XRCC2, XRCC3 and NBN SNPs were shown on agarose electrophoresis. (TIF) [file pone.0020055.s001.tif]
